# Efficacy and Safety of Chinese Patent Medicine for the Prevention and Treatment of Radiotherapy and Chemotherapy-Induced Oral Mucositis: A Systematic Review and Meta-Analysis

**DOI:** 10.3389/fphar.2022.812085

**Published:** 2022-03-28

**Authors:** Yufei Xie, Xin Fang, Hong Hua, Peiru Zhou

**Affiliations:** Department of Oral Medicine, Peking University School and Hospital of Stomatology, National Center of Stomatology, National Clinical Research Center for Oral Diseases, National Engineering Laboratory for Digital and Material Technology of Stomatology, Beijing, China

**Keywords:** Chinese patent medicine, oral mucositis, radiotherapy, chemotherapy, systematic review, meta-analysis

## Abstract

**Background:** Radiotherapy and chemotherapy-induced oral mucositis can affect cancer patients’ quality of life, even necessitate cancer therapy and influence prognosis. Chinese patent medicines (CPMs) have been widely used as complementary alternative medicines for the prevention and treatment of oral mucositis, and their efficacy and safety require further evaluation. Therefore, this study was conducted to provide references for clinical practice.

**Methods:** Ten databases were searched electronically and manually to identify randomized controlled trials (RCTs) from their inception to August 2021, concerning the prevention and treatment of radiotherapy and chemotherapy-induced oral mucositis with CPMs. The prevalence, pain level, and the severity of radiotherapy and chemotherapy-induced oral mucositis, as well as the effectiveness rate and adverse effects of CPMs, were set as the outcome criteria. The assessment criteria of the Cochrane Handbook were used to determine study quality and bias, and meta-analysis was conducted using Review Manager 5.4.1 software.

**Results:** A total of 2,312 cases from 27 RCTs were included. Most studies were considered to have a low or unclear risk of bias. More research is available on the use of CPMs in the prevention of radiotherapy and chemotherapy-induced oral mucositis than in its treatment. As for the prevention, it was proved that CPMs could significantly reduce the prevalence of radiotherapy and chemotherapy-induced oral mucositis, especially for the severe types, and decrease pain levels (*p* < 0.05). For treatment, CPMs could alleviate the symptoms, promote the healing of ulceration in radiotherapy and chemotherapy-induced oral mucositis, and thus improve the efficiency of clinical treatment (*p* < 0.05). The results of subgroup analyses were mainly consistent with the above results. The adverse effects of CPMs mainly included gastrointestinal reactions and bitter taste, and no serious adverse events were reported.

**Conclusions:** This systematic review and meta-analysis indicated CPMs might be effective for the prevention and treatment of radiotherapy and chemotherapy-induced oral mucositis through reducing the prevalence, decreasing the occurrence of severe types, alleviating the symptoms, and promoting the healing of ulceration. However, due to the limited number of eligible studies and the publication bias, more high-quality, double-blinded, placebo-controlled RCTs are still needed in future research.

**Systematic Review Registration**: [https://inplasy.com/], identifier [INPLASY2021100100].

## 1 Introduction

Oral mucositis is an inflammation of the mucosa with a burning or tingling sensation. It is a common toxicity associated with both chemotherapy and head and neck radiation used for the treatment of cancer (Scully et al., 2006; Lalla et al., 2014a). Although many therapeutic strategies such as systemic chemotherapy and radiotherapy have improved survival rates for tumors, these treatments also cause several side effects, including a high risk of developing oral mucositis (Lalla et al., 2014b). Mucositis can be divided by treatment modality of cancer patients: radiotherapy to head and neck (H&N) cancer-induced (RT-induced), radiotherapy and chemotherapy to H&N cancer-induced (RT-CT-induced), chemotherapy-induced (CT-induced), and hematopoietic stem cell transplantation–induced (HSCT-induced). Mucositis has been consistently reported to occur in at least 75% of these cancer patients (Scully et al., 2006). RT-induced oral mucositis occurs in almost all patients who are treated for cancers of H&N; Grade 3 and 4 oral mucositis occurs in 56% (Jones et al., 2006; Maria et al., 2017). About 40% of patients treated with standard chemotherapy develop CT-induced oral mucositis. The situation can be even worse for the combining of different chemotherapeutic drugs, and the likelihood of oral mucositis increases with the number of chemotherapy cycles (Naidu et al., 2004; Scully et al., 2006). Mucositis presents as erythema and ulceration of the oral mucosa and is pathologically characterized by atrophy of the squamous epithelium, vascular damage, inflammatory infiltration, and ulceration. It causes severe discomfort and impairs patients’ ability to swallow, eat, and talk. The pain of oral mucositis increases the use of opioid analgesics, impairs nutritional intake, affecting the quality of life (Elting et al., 2008; Jensen and Peterson, 2014). Severe mucositis can necessitate cancer therapy, causing interruptions and/or dose reductions in cancer treatment protocols, and even negatively influence prognosis (Peterson et al., 2015).

Numerous strategies have been recommended by the Multinational Association of Supportive Care in Cancer/International Society of Oral Oncology (MASCC/ISOO) for the prevention and treatment of radiotherapy and chemotherapy-induced oral mucositis. In the latest guideline conducted by the group in 2020, some natural agents, including herbal medicines, are included in the analysis. But due to the conflicting evidence, they are listed as having “no guideline possible” (Yarom et al., 2019; Yarom et al., 2020). Therefore, it is of great significance to assess their effects and thus lead to their appropriate clinical use. Chinese patent medicines (CPMs) are modern pharmaceutical preparations of traditional Chinese medicine (TCM) extracts, such as TCM pills, capsules, and injections, made mainly according to a standardized formulation of Chinese herbal medicines. The registration of CPMs is authorized by the China Food and Drug Administration (CDFA) and National Administration of Traditional Chinese Medicine (NATCM), with reference to laws such as *Drug Administration Law of the People’s Republic of China* and *Provision for Drug Registration* (Zhang, 2018). According to the TCM theory, CPMs with heat-clearing and detoxicating effects have become ideal options in the prevention and treatment of heat toxin syndrome, including redness, swelling, ulceration, and pain, which are also the main features of oral mucositis. In addition, unlike synthetic medicines, naturally originated CPMs are generally perceived to have a wider range of sources, fewer side effects, and multiple pharmacological targets (Lin et al., 2021). Therefore, CPMs are in wide clinical use to prevent or treat radiotherapy and chemotherapy-induced oral mucositis in China as important complementary alternative medicines (Zheng et al., 2011). However, as most articles relevant to CPMs are published in Chinese, their number and quality may be underestimated by clinicians and scientists outside China. Moreover, there has been no systematic review and meta-analysis evaluating the effectiveness of CPMs for radiotherapy and chemotherapy-induced oral mucositis currently. Therefore, with the inclusion of Chinese and English publications, a comprehensive assessment of the efficacy and safety of CPMs for the prevention and treatment of radiotherapy and chemotherapy-induced oral mucositis is conducted in this study to provide basic information for clinical practice.

## 2 Materials and Methods

### 2.1 Protocol and Registration

This study was conducted and presented according to the Preferred Reporting Items for Systematic Reviews and Meta-Analyses (PRISMA) (Page et al., 2021). The research protocol has been registered in Inplasy (https://inplasy.com/) (Registration NO: INPLASY2021100100).

### 2.2 Search Strategy

We selected all clinical trials in English or Chinese that focused on CPMs for the prevention and treatment of chemotherapy and radiotherapy-induced oral mucositis in the Pubmed, Web of Science, embase, CNKI, CBM, CQVIP, Wan-Fang, Ovid, Current Controlled Trials, and Cochrane Library. These databases were searched up to August 2021. The reference lists of the articles were also searched. The following search terms were used individually or combined: Chinese Patent Medicine, Oral Mucositis, Radiotherapy, Chemotherapy, as well as their synonyms. The searches were conducted by two independent investigators (X Fang and PR Zhou).

### 2.3 Inclusion and Exclusion Criteria

Studies that met all of the following five criteria were included in this review.

1) Participants: patients were diagnosed with cancer and received radiotherapy, chemotherapy, or both, with no restriction on the cancer location and nature.
2) Interventions: the treatment groups were treated with systemically or topically administered CPMs without the combination of Western medicines.
3) Control: the control groups were with no treatment, or treated with placebos and agents including vitamin preparations, normal saline, and antiseptic or anesthetic mouthwashes.
4) Outcome: the studies must have reported at least one of the following primary or secondary outcomes: the primary outcomes included the prevalence of oral mucositis, the level of pain, the grading of oral mucositis, and the effectiveness rate; the secondary outcomes were the cure days of oral mucositis and the adverse effects.
5) Types of studies: randomized controlled trials (RCTs).

The following types of studies were excluded.

1) Publication in a language other than English or Chinese.
2) Duplicate publications reporting the same groups of participants.
3) Case Reports, Reviews, Workshop Summaries, and Observational Studies

### 2.4 Data Collection and Quality Assessment

The literature search, data collection, and the assessment of the risk of bias were carried out by two independent researchers (PR Zhou and X Fang) using the criteria from the *Cochrane Reviewers’ Handbook 6.0* as follows (Higgins et al., 2019). 1) Search results from the ten databases were imported into the reference management software EndNote X9. 2) Duplicated and irrelevant studies were removed by screening the title and abstract. 3) Full papers were obtained for all possibly relevant trials. 4) Studies meeting the inclusion criteria were identified. 5) The following data were collected: title, article source, year of publication, author(s), study size, sample size, methodological details, cancer type, mucositis type (i.e., related to RT, RT-CT, CT, or HSCT), the intervention of the treatment and control groups, outcome measures, and adverse effects. Disagreements were resolved by discussion, and the consensus was achieved through additional reviewers (H Hua).

The risk of bias was evaluated by Review Manager 5.4.1 software according to seven domains: random sequence generation (selection bias), allocation concealment (selection bias), masking of participants and personnel (performance bias), masking of outcome assessment (detection bias), incomplete outcome data (attrition bias), selective reporting (reporting bias), and other bias.

### 2.5 Data Synthesis and Statistical Analysis

\Statistical analyses were conducted using Review Manager 5.4.1 software. Subgroup analysis was undertaken for systemically and topically used CPMs. The estimate of the intervention effect was expressed as the odds ratios (OR) together with the 95% confidence interval (CI) and plotted on a forest plot. The *I* ^*2*^ test was performed to evaluate the heterogeneity of the studies and ranged from 0 to 100%; *I* ^*2*^ values of 25 and 50% were used as cutoffs for modest and high heterogeneity (Ioannidis, 2008). If no significant heterogeneity was found, a fixed-effects model was used to calculate the combined OR. Otherwise, a random-effects model was used. A *p* value <0.05 was regarded as statistically significant. Funnel plots were used to evaluate underlying publication bias.

## 3 Result

### 3.1 Study Selection and Characteristics of the Included Studies

**FIGURE 1:**
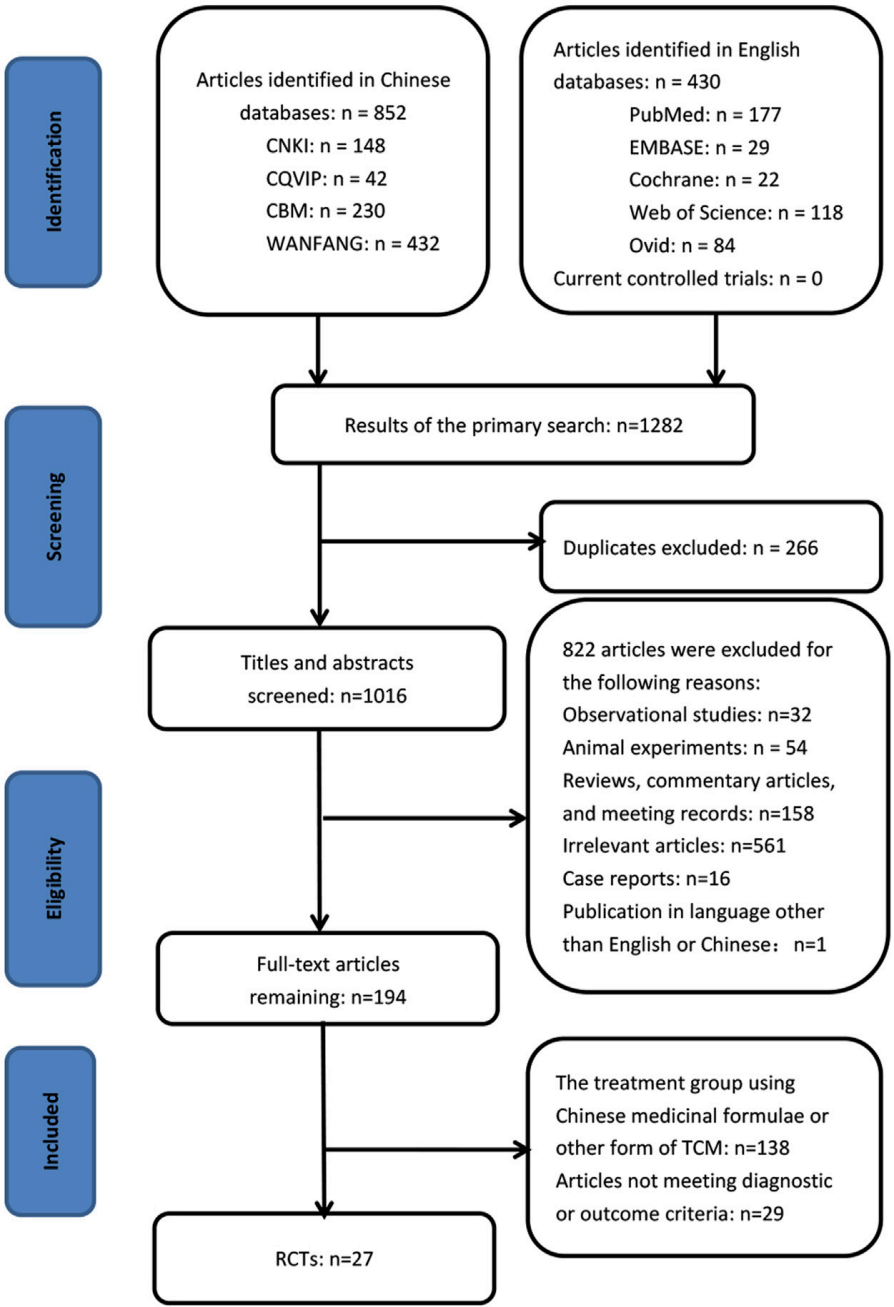
Systematic review flow diagram.

By searching the ten electronic databases, we initially identified 1,282 publications. Of these, 266 studies were excluded as duplicates, 561 were irrelevant articles, 260 were not RCTs or controlled clinical trials, and one was in a language other than English or Chinese. After we read the full papers of the remaining 194 articles, 138 were excluded because the treatment groups used Chinese medicinal formulas or other forms of Traditional Chinese Medicines rather than the standardized CPM, and 29 were excluded because they did not meet the diagnostic or outcome criteria. Finally, 27 trials were included in the final review: two studies were published in English, and 25 studies were in Chinese (Figure 1). The characteristics of the included 27 studies are shown in Tables 1, 2.

**TABLE 1:** Characteristics of the RCTs for the prevention of oral mucositis.

No	Study	Cancer Type	Mucositis Type	Sample Size (Treatment/Control)	Age of the Patients (Treatment/Control)	Intervention		Outcome measure^#^	Adverse Event
						Treatment	Control		
1	Xu *et al.* (2013)	Nasopharyngeal carcinoma	RT-CT-related	30/30	46.03 ± 14.24/46.57 ± 13.88	Zhenhuang pills 0.4 g orally, 3 times per day	Mouth rinsed with mouth wash containing lidocaine, dexamethasone, and gentamycin, with no information about the dose and frequency	1	Two cases suffered loose stools during medication
2	Wang W. M. *et al.* (2010)	Nasopharyngeal carcinoma	RT-CT-related	30/30	46.3 ± 11.5/45.3 ± 13.0	Yangyin Shengxue Mixture 50 ml orally, 1 time per day, 3 days before radiotherapy till 1 week after	Mouth rinsed with Tinidazole gargle, 6–8 times per day, with no information about the dose	1, 4	Three patients developed mild nausea or diarrhea
3	Dong *et al.* (2017)	Nasopharyngeal carcinoma	RT-related	40/40	18–80/18–80	Xihuang Capsules 1.50 g orally, 2 times per day	No treatment	1	Not mentioned
4	Li, (2011)	Head and neck cancer	RT-related	32/32	53.8 ± 19.1/44.1 ± 7.8	Kouyanqing Granules 20 g, 2 times per day	No treatment	1	Not mentioned
5	Ding and Xu, (2015)	Nasopharyngeal carcinoma	RT-related	18/18	19–70/19–70	Mailuoning injection 20 ml, intravenously guttae, 1 time per day	No treatment	1	Not mentioned
6	Yan *et al.* (2017)	Head and Neck Cancer	RT-related	61/61	46.11 ± 10.86/46.16 ± 9.96	Kouyanqing Granules 20 g dissolved in 50 ml lukewarm boiled water orally, 2 times per day, using 7 weeks from 2 to 3 days before radiotherapy	Mouth rinsed with 0.12% chlorhexidine gargle 10–20 ml, 2 times per day, using 7 weeks from 2 to 3 days before radiotherapy	1, 4	One patient developed mild nausea and vomiting
7	Wang *et al.* (2017)	Not mentioned	CT-related	43/42	47.07 ± 9.62/44.83 ± 9.97	Kouyanqing Granules 20 g dissolved in 50 ml lukewarm boiled water orally, 2 times per day	Mouth rinsed with 0.12% chlorhexidine gargle 10–20 ml, 2 times per day	1, 4	Not mentioned
8	Hua et al. (2011)	Nasopharyngeal carcinoma	RT-CT-related	28/23	Over 18/over 18	Kanglaite injection 200 ml, intravenously guttae, 1 time per day	Placebo	3	Not mentioned
9	Ding and Gu, (2011)	Colorectal cancer	CT-related	23/27	27–76/27–76	Kangfuxin solution 10 ml orally, 3 times per day	No treatment	1	Not mentioned
10	Dai and Tang, (2010)	Nasopharyngeal carcinoma	RT-CT-related	29/29	29–71/27–69	Kangai injection 40 ml added with 5% glucose solution or 0.9% normal saline 250 ml intravenously guttae, 1 time per day, 5 days a week, until the end of radiotherapy	No treatment	1	Not mentioned
11	Gao, (2004)	Cancer of the digestive tract, lung, and breast, sarcoma, and lymphoma	CT-related	90/90	18–82/18–82	Huangqi injection 30 ml added with 5% glucose solution 250 ml intravenously guttae, 1 time per day, using 3 days before chemotherapy, in total 20 days	No treatment	1	Not mentioned
12	Zhao and Li, (2011)	Gastric carcinoma	CT-related	30/26	35–72/35–72	Tianzhicao capsule, 5 capsules orally, 3 times per day	No treatment	1	Not mentioned
13	Wang Y. Z. *et al.* (2010)	Gastric carcinoma	CT-related	20/20	40–75/40–75	Diluted Cinobufotalin injection 10–20 ml intravenously guttae, 1 time per day for 10 days for a period of 2 weeks as a cycle, using 8 cycles in total	No treatment	3	Not mentioned
14	Lian *et al.* (2017)	Nasopharyngeal carcinoma	RT-related	44/44	Over 18/over 18	Compound Kushen injection, 20 ml intravenously guttae, 1 time per day from the first day of radiotherapy, 5 days a week, until the end of radiotherapy	The riboflavin injection 20 mg intravenous drip once a day from the first day, 5 days a week, until the end of radiotherapy	1, 4	Bitter taste appeared in 2 patients and mild diarrhea in 1 patient
15	Tao and Xu, (2013)	Colon cancer	CT-related	74/74	60.1 ± 7.9/60.4 ± 8.9	Compound Kushen injection, 15 ml intravenously guttae, 1 time per day, 14 days before chemotherapy, 5 weeks for one cycle and 3 cycles for one course of treatment	No treatment	1	Not mentioned
16	Zhan and Zhang, (2017)	Rectal cancer	CT-related	64/64	62.4 ± 11.8/62.1 ± 11.6	Compound Kushen injection, 12 ml intravenously guttae, 1 time per day, 14 days for 1 cycle, 2 cycles for 1 course of treatment, and a total of 4 courses	No treatment	3	Not mentioned
17	Jiang, (2014)	Non-small cell lung cancer	CT-related	39/39	57.26 ± 7.92/58.49 ± 8.19	Babaodan capsule, 2 capsules orally, 3 times per day, for 3 weeks as a cycle, 2 cycles for one course of treatment	No treatment	1	Not mentioned
18	Zheng *et al.* (2018)	Nasopharyngeal carcinoma	RT-CT-related	119/119	47.63 ± 10.28/48.86 ± 9.36	Shuanghua Baihe tablets, 4 tablets orally, 3 times per day, for 7 weeks	Placebo tablets, 4 tablets orally, 3 times per day, for 7 weeks	1, 3	Four patients suffered mild or moderate gastrectasia reactions
19	Lin et al. (2014)	Head and neck cancer	RT-related	38/35	50.87 ± 14.56/54.03 ± 16.10	Tianwang Buxin Mini-Pills 3 g orally, 3 times per day, using from the initiation of radiotherapy until 1 month after completion	Placebo	3	Not mentioned
20	Wei *et al.* (2011)	Nasopharyngeal carcinoma	RT-CT-related	30/30	Over 18/over 18	Compound Kushen injection 20 ml intravenously guttae, 1 time per day, 15 days for one cycle, repeating the cycle after 5 days interval, using 3 cycles for one course of treatment	No treatment	1	Not mentioned
21	Xia *et al.* (2015)	Head and neck cancer	RT-related	25/25	18–76/18–76	10 ml Kangfuxin solution and 10 ml normal saline were used for atomization inhalation once a day from the beginning of radiotherapy until the end	Rinse with 20 ml normal saline 4 times a day	1	Not mentioned
22	Bai *et al.* (2017)	Nasopharyngeal carcinoma	RT-CT-related	107/108	46.3 ± 11.0/48.0 ± 10.0	Gargle with 10 ml Kangfuxin solution, 3–5 min each time, and then take orally, 3 times per day	Gargle with 10 ml 5-times diluted compound borax, 3–5 min each time 3 times per day	1, 3	Not mentioned
23	Wang and Ma, (2014)	Head and neck cancer	RT-related	30/30	55.43 ± 9.12/53.86 ± 9.01	Gargle with 10 ml Kangfuxin solution and then take orally, 4 times per day	Gargle with 10 ml normal saline 4 times per day	1	Not mentioned

Notes: #:1—effective for mucositis severity; 2—effective for mucositis duration; 3—the prevalence of oral mucositis; 4—effective for pain severity; 5—effective for pain duration; 6—the effective rate.

**TABLE 2:** Characteristics of the RCTs for the treatment of oral mucositis.

No	Study	Cancer Type	Mucositis Type	Sample Size (Treatment/Control)	Age of the Patients (Treatment/Control)	Intervention (Course: 7–14 days)		Outcome measure^#^	Adverse Event
						Treatment	Control		
24	Zhao et al. (2010)	Nasopharyngeal carcinoma	RT-related	35/35	43.87 ± 4.27/42.53 ± 4.91	Gargle with 5–10 ml Xipayi mouth rinse, 2–3 min each time, 3–5 times per day	Gargle with 5–10 ml 5-times diluted compound borax solution, 2–3 min each time, 3–5 times per day	1, 6	Not mentioned
25	Chen et al. (2011)	Cancer of the digestive tract and nasopharyngeal carcinoma	CT-related	30/30	36–70/34–70	Apply Sai wei ‘an directly applied to the affected area, 5 times per day, 7 days of treatment	B complex vitamin tablets, 2 tablets orally, 3 times per day	2, 6	Not mentioned
26	Gong and A, (2006)	acute leukemia	CT-related	30/20	15–70/15–64	Apply oral ulcer powder to the affected area, 3 times per day	Gargle with 20 ml 0.12% chlorhexidine gargle, 3 times per day	1, 2	Not mentioned
27	Li and Tan, (2012)	Cancer of the digestive tract, lung, and breast, nasopharyngeal carcinoma, osteosarcoma, and lymphoma	CT-related	26/26	18–64/18–64	Gargle with 10–15 ml Kangfuxin solution, 3 or 4 times per day	Rinse with normal saline, 3–5 times per day	6	Not mentioned

Notes: #:1—effective for mucositis severity; 2—effective for mucositis duration; 3—the prevalence of oral mucositis; 4—effective for pain severity; 5—effective for pain duration; 6—the effective rate.

### 3.2 Risk of Bias Assessment

**FIGURE 2:**
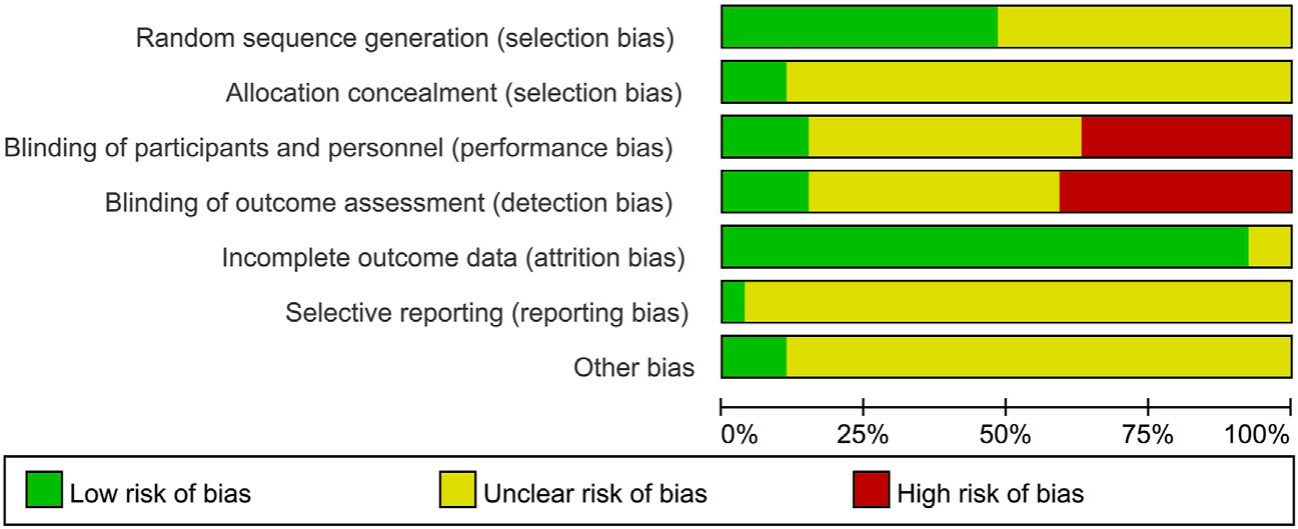
Risk of bias: review authors’ judgments about each risk of bias presented as percentages across all included studies.

**FIGURE 3:**
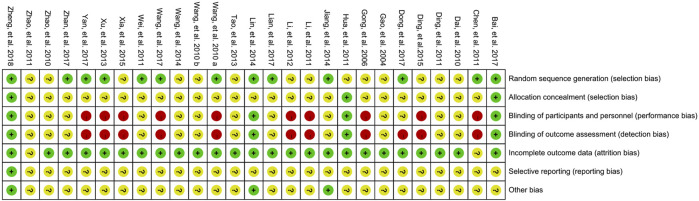
Risk of bias summary: review authors’ judgments about each risk of bias for each included study (green, low risk of bias; yellow, unclear risk of bias; red, high risk of bias).

The risk of bias was assessed according to the Cochrane quality assessment criteria. The assessment is presented graphically by study (Figure 2) or by domain over all of the studies (Figure 3). All twenty-seven studies were RCTs. For random sequence generation, thirteen studies (Wang W. M. et al., 2010; Chen et al., 2011; Wei et al., 2011; Xu et al., 2013; Jiang, 2014; Lin et al., 2014; Bai et al., 2017; Dong et al., 2017; Lian et al., 2017; Wang et al., 2017; Yan et al., 2017; Zhan and Zhang, 2017; Zheng et al., 2018) were rated as low risk bias; For allocation concealment, three studies (Hua et al., 2011; Bai et al., 2017; Zheng et al., 2018) were rated as low risk bias; For blinding of participants and personnel, four studies (Hua et al., 2011; Lin et al., 2014; Bai et al., 2017; Zheng et al., 2018) were rated as low risk bias; For blinding of outcome assessment, four studies (Hua et al., 2011; Lin et al., 2014; Bai et al., 2017; Zheng et al., 2018) were rated as low risk bias; For imcomplete outcome data, only one study (Zhao et al., 2010) was rated as having an unclear risk of bias; For selective supporting and other bias, one (Zheng et al., 2018) and three studies (Jiang, 2014; Lin et al., 2014; Zheng et al., 2018) were rated as having an low risk of bias, respectively.

### 3.3 Effectiveness of CPMs for the prevention of radiotherapy and chemotherapy-induced oral mucositis

We examined 23 studies (Gao, 2004; Dai and Tang, 2010; Wang W. M. et al., 2010; Wang Y. Z. et al., 2010; Ding and Gu, 2011; Hua et al., 2011; Li, 2011; Wei et al., 2011; Zhao and Li, 2011; Tao and Xu, 2013; Xu et al., 2013; Jiang, 2014; Lin et al., 2014; Wang and Ma, 2014; Ding and Xu, 2015; Xia et al., 2015; Bai et al., 2017; Dong et al., 2017; Lian et al., 2017; Wang et al., 2017; Yan et al., 2017; Zhan and Zhang, 2017; Zheng et al., 2018) of the effectiveness of CPMs in the prevention of radiotherapy and chemotherapy-induced oral mucositis, namely, Zhenhuang pills, Yangyin Shengxue Mixture, Xihuang capsules, Kouyanqing Granules, Mailuoning injection, Kanglaite injection, Kangfuxin solution, Kangai injection, Huangqi injection, Tianzhicao capsules, Cinobufotalin injection, Compound Kushen injection, Babao capsules, Shuanghua Baihe tablets, and Tianwang Buxin Mini-Pills. The comparators were drug-free controls, placebos, or agents including vitamin preparations, normal saline, and mouthwashes. The prevalences, moderate and severe pain, as well as Grade 3 and 4 radiotherapy and chemotherapy-induced oral mucositis, were pooled and meta-analyzed.

#### 3.3.1 CPMs Reduced the Prevalence of Radiotherapy and Chemotherapy-Induced Oral Mucositis

**FIGURE 4:**
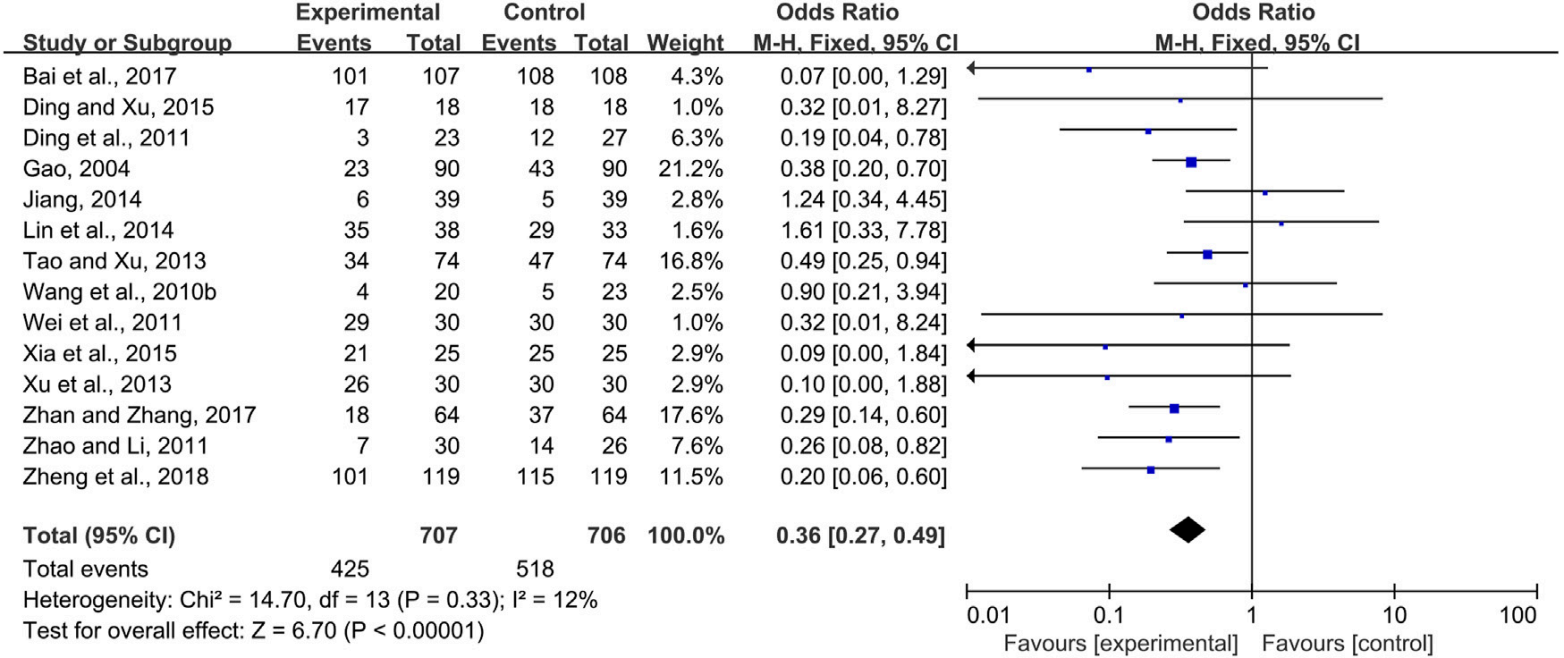
The efficacy of CPMs for the prevalence of radiotherapy and chemotherapy-induced oral mucositis.

Fourteen trials (Gao, 2004; Wang Y. Z et al., 2010; Ding and Gu, 2011; Wei et al., 2011; Zhao and Li, 2011; Tao and Xu, 2013; Xu et al., 2013; Jiang, 2014; Lin et al., 2014; Ding and Xu, 2015; Xia et al., 2015; Bai et al., 2017; Zhan and Zhang, 2017; Zheng et al., 2018) comparing the prevalence of radiotherapy and chemotherapy-induced oral mucositis were demonstrated in the forest plot. Due to fairly low heterogeneity among the trials (*I* ^2^ = 18%), a fixed-effects model was chosen for the meta-analysis. The results showed that CPMs were superior to controls in reducing the prevalence of radiotherapy and chemotherapy-induced oral mucositis (pooled OR = 0.36, 95% CI = 0.27–0.49, *p* < 0.00001; Figure 4).

**FIGURE 5:**
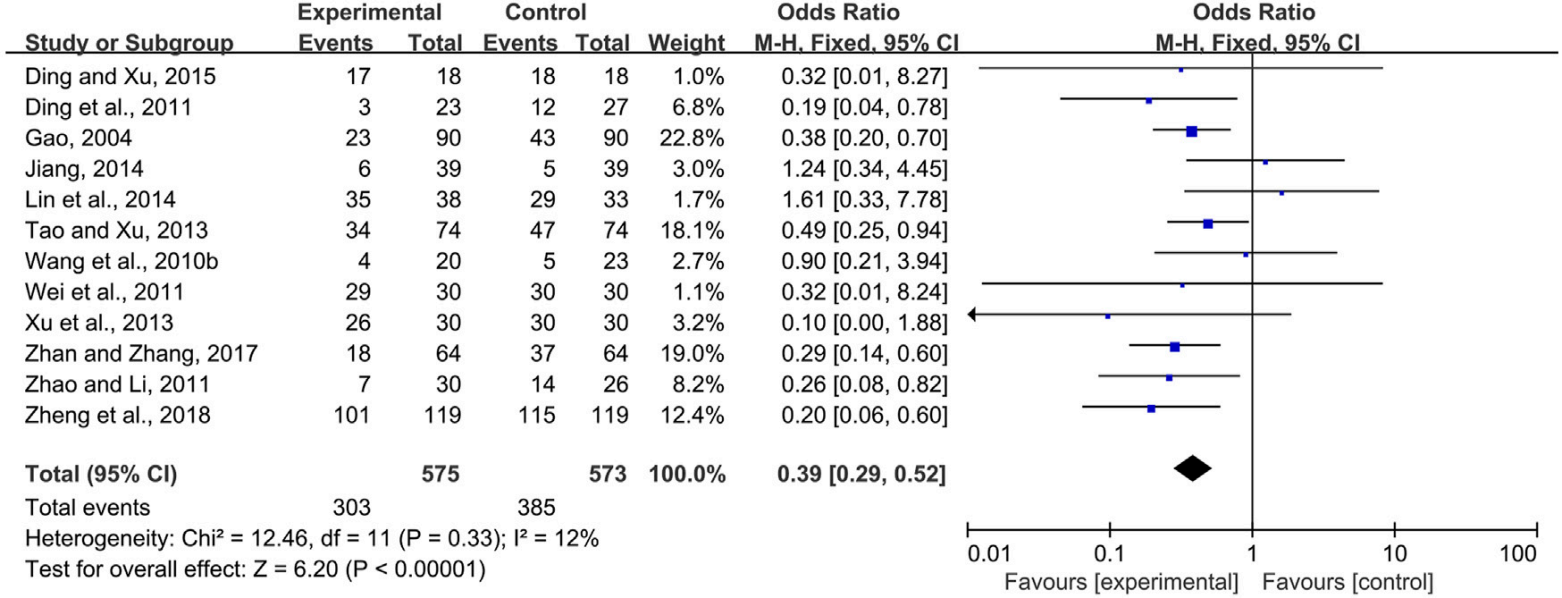
The efficacy of systemically used CPMs for the prevalence of radiotherapy and chemotherapy-induced oral mucositis.

**FIGURE 6:**
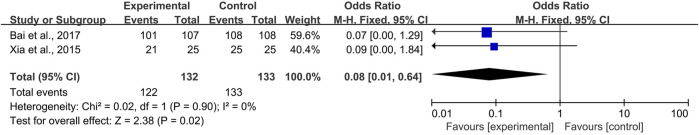
The efficacy of topically used CPMs for the prevalence of radiotherapy and chemotherapy-induced oral mucositis.

Systemic and topical use of CPMs were further analyzed separately. The results of meta-analysis using fixed-effects models (*I* ^*2*^ = 12 and 0%, respectively) showed that, whether used systemically or topically, CPMs were superior to controls in reducing the prevalence of radiotherapy and chemotherapy-induced oral mucositis (pooled OR = 0.39, 95% CI = 0.29–0.52, *p* < 0.00001; pooled OR = 0.08, 95% CI = 0.01–0.64, *p* = 0.02; respectively) (Figures 5, 6).

#### 3.3.2 CPMs Decreased the Pain Level in Radiotherapy and Chemotherapy-Induced Oral Mucositis

**FIGURE 7:**
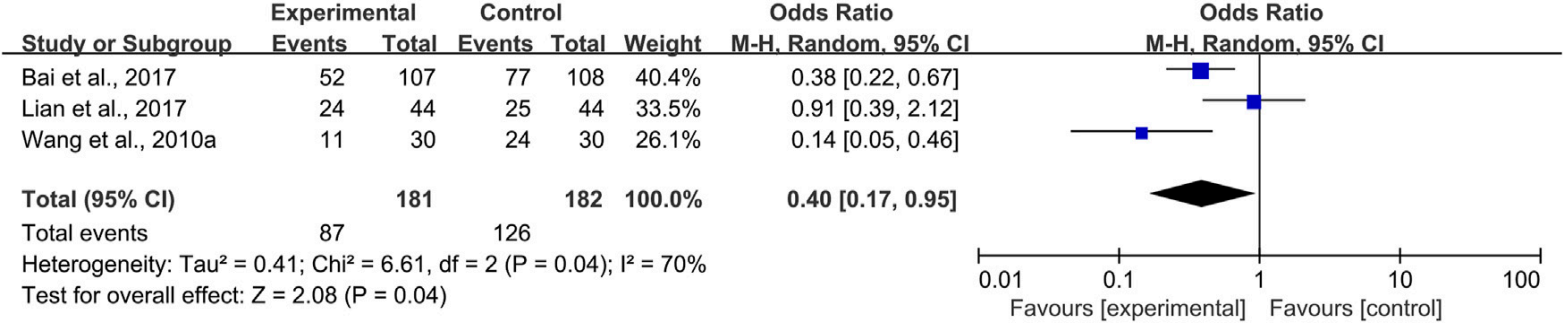
The efficacy of CPMs for the prevalence of moderate and severe pain in radiotherapy and chemotherapy-induced oral mucositis.

Two trials of systemic use (Wang W. M. et al., 2010; Lian et al., 2017) and one trial (Bai et al.,2017) of topical use compared the pain level of the CPM groups and control groups. Moderate and severe pain scored above four on the 0–10 numerical rating scale or visual analog scale. Due to heterogeneity among the trials (*I* ^2^ = 70%), a random-effects model was chosen for the meta-analysis. The results showed that CPMs were superior to controls in reducing the prevalence of moderate and severe pain (pooled OR = 0.40, 95% CI = 0.17–0.95, *p* = 0.04; Figure 7). An analysis of systemic and topical subgroups was not conducted because of the limited number of studies.

#### 3.3.3 CPMs Reduced the Severity of Radiotherapy and Chemotherapy-Induced Oral Mucositis

**FIGURE 8:**
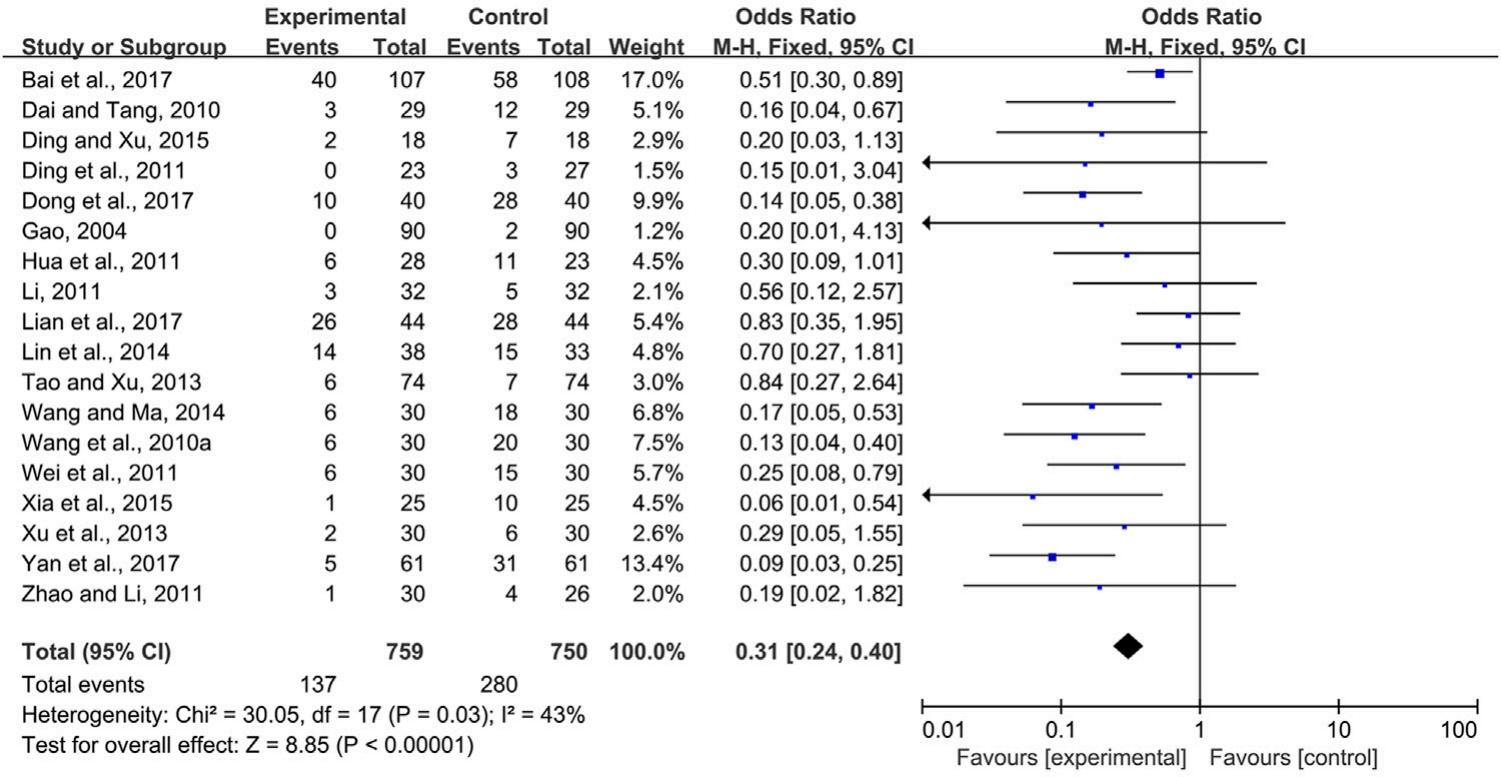
The efficacy of CPMs for reducing the severity of radiotherapy and chemotherapy-induced oral mucositis.

Eighteen trials (Gao, 2004; Dai and Tang, 2010; Wang W. M. et al., 2010; Ding and Gu, 2011; Hua et al., 2011; Li, 2011; Wei et al., 2011; Zhao and Li, 2011; Tao and Xu, 2013; Xu et al., 2013; Lin et al., 2014; Wang and Ma, 2014; Ding and Xu, 2015; Xia et al., 2015; Bai et al., 2017; Dong et al., 2017; Lian et al., 2017; Yan et al., 2017) compared the prevalence of severe radiotherapy and chemotherapy-induced oral mucositis in the experimental and control groups, i.e., Grade 3 and 4 radiotherapy and chemotherapy-induced oral mucositis according to the criteria of the World Health Organization or toxicity criteria of the Radiation Therapy Oncology Group (RTOG) (Miller et al., 1981; Cox et al., 1995). Due to modest heterogeneity among the trials (*I* ^*2*^ = 43%), a fixed-effects model was chosen for the meta-analysis. The results showed that CPMs were superior to controls in reducing the severity of radiotherapy and chemotherapy-induced oral mucositis (pooled OR = 0.31, 95% CI = 0.24–0.40, *p* < 0.00001; Figure 8).

**FIGURE 9:**
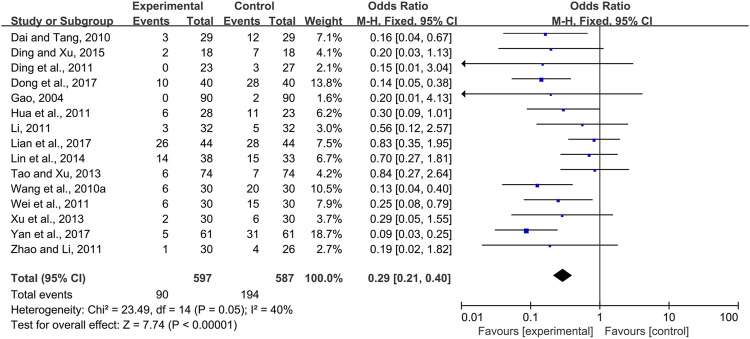
The efficacy of systemically used CPMs for the prevalence of severe radiotherapy and chemotherapy-induced oral mucositis.

**FIGURE 10:**
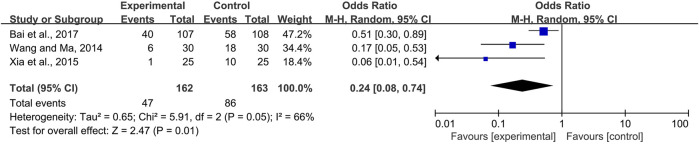
The efficacy of topically used CPMs for the prevalence of severe radiotherapy and chemotherapy-induced oral mucositis.

The effectiveness of systemic and topical use of CPMs was further analyzed separately. The results of meta-analysis using a fixed-effects model and a random-effects model (*I* ^*2*^ = 40 and 66%, respectively) showed that, whether used systemically or topically, CPMs were superior to controls in reducing the severity of radiotherapy and chemotherapy-induced oral mucositis (pooled OR = 0.29, 95% CI = 0.21–0.40, *p* < 0.00001; pooled OR = 0.24, 95% CI = 0.08–0.74, *p* = 0.01 respectively) (Figures 9, 10).

### 3.4 Effectiveness of CPMs for the treatment of radiotherapy and chemotherapy-induced oral mucositis

**FIGURE 11:**
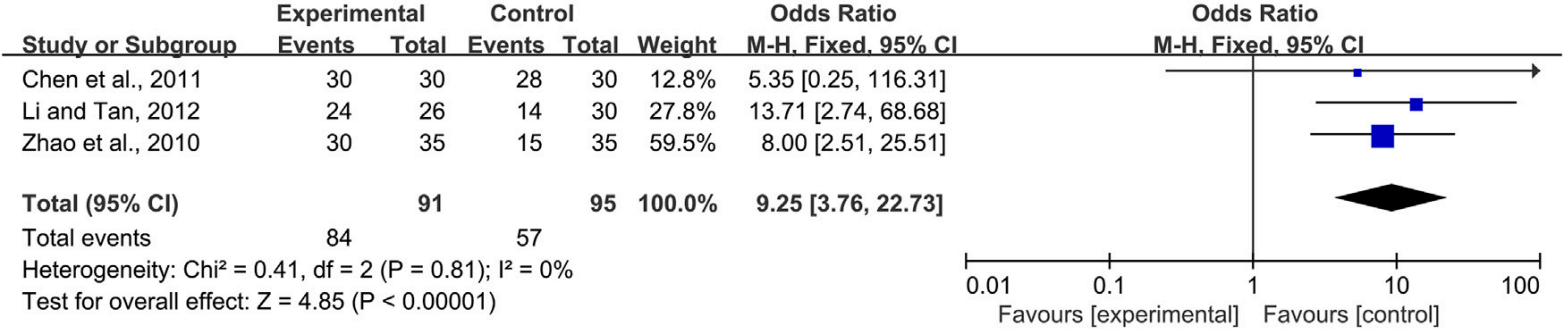
The efficacy of CPMs for the effective rate of radiotherapy and chemotherapy-induced oral mucositis.

Four studies (Gong and A, 2006; Zhao et al., 2010; Chen et al., 2011; Li and Tan, 2012) compared the effects of CPMs, including Xipayi mouth rinse, Sai wei’an, oral ulcer powder, and Kangfuxin solution in the treatment of radiotherapy and chemotherapy-induced oral mucositis; the comparators were the compound borax solution, B complex vitamin tablets, 0.12% chlorhexidine gargle, and normal saline, respectively. Three trials (Zhao et al., 2010; Chen et al., 2011; Li and Tan, 2012) reported the effective rate of radiotherapy and chemotherapy-induced oral mucositis, which means the percentage of oral mucositis patients with pain and/or ulcer relief. Due to no heterogeneity among the trials (*I* ^*2*^ = 0%), a fixed-effects model was chosen for the meta-analysis. The results showed that CPMs were superior to controls in increasing the effective rate (pooled OR = 9.25, 95% CI = 3.76–22.73, *p* < 0.00001; Figure 11).

Moreover, there was evidence from one trial (Gong and A, 2006) that oral ulcer powder was more effective in shortening the cure days of radiotherapy and chemotherapy-induced oral mucositis (*p* < 0.05) than chlorhexidine gargle. However, no difference between the two groups was found with regard to the average grade of radiotherapy and chemotherapy-induced oral mucositis after treatment (*p* > 0.05).

### 3.5 Safety Assessment

Five trials (Wang W. M. et al., 2010; Xu et al., 2013; Lian et al., 2017; Yan et al., 2017; Zheng et al., 2018) reported adverse effects. The CPMs included Zhenhuang pills, Yangyin Shengxue Mixture, Kouyanqing Granules, Compound Kushen injection, and Shuanghua Baihe tablets. The most frequently reported adverse effects were gastrointestinal reactions including diarrhea, loose stools, nausea, and vomiting, which involved Yangyin Shengxue Mixture, Kouyanqing Granules, Compound Kushen injection, Shuanghua Baihe tablets, and Zhenhuang pills (Wang W. M. et al., 2010; Lian et al., 2017; Yan et al., 2017; Zheng et al., 2018). Bitter taste was reported in two patients treated by Compound Kushen injection during medication (Lian et al., 2017). The adverse effects were generally mild and improved after the patients discontinued the medicine. No serious adverse events were reported.

### 3.6 Bias Analysis

**FIGURE 12:**
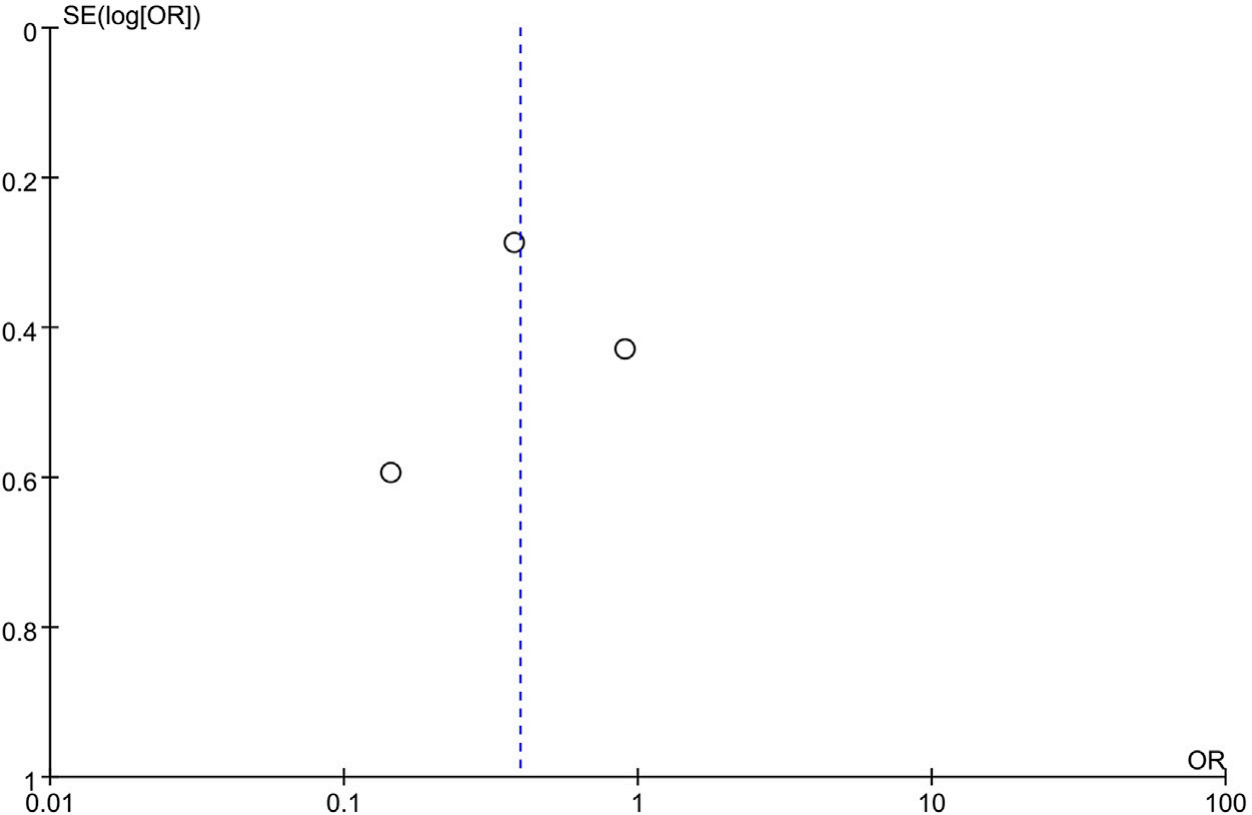
Funnel plot of studies evaluating the prevalence rate of moderate and severe pain between CPMs groups and control groups.

Concerns about biases affecting the outcomes of this study existed due to the low quality of the RCTs reviewed for analysis. Moreover, because of the substantial heterogeneity among the included studies evaluating the prevalence of moderate and severe pain (*I* ^2^ = 70%), it is inappropriate to confirm the above results directly. Therefore, the publication bias was assessed. The shape of the funnel plot showed no evidence of asymmetry (Figure 12).

## 4 Discussion

In China, more and more patients receiving radiotherapy and chemotherapy choose CPMs to prevent or treat radiotherapy and chemotherapy-induced oral mucositis, but the application of CPMs is basically experience-based. Therefore, we tried to build its missing link to the evidence-based medical sciences in the present study. From the review, we found that CPMs were more frequently used in the prevention than the treatment of radiotherapy and chemotherapy-induced oral mucositis. That means the use of CPMs was usually begun before the onset of the disease, which may reduce the progression of the disease and thus had the effect of reducing the severity of radiotherapy and chemotherapy-induced oral mucositis. By meta-analysis, it was proved that CPMs could significantly reduce the prevalence, pain level, and severity of radiotherapy and chemotherapy-induced oral mucositis. As for the treatment, CPMs were effective in alleviating the symptoms, reducing the cure days, and thus improving the efficiency of clinical treatment for radiotherapy and chemotherapy-induced oral mucositis. We also found that the systemic administration of CPMs was more frequent than topical. Systemic CPMs could prevent and treat radiotherapy and chemotherapy-induced oral mucositis mainly by reducing the prevalence and severity of oral mucositis, and topical CPMs could play a role primarily through relieving the symptoms, promoting the healing of ulceration, and reducing the severity of oral mucositis.

According to the TCM theory, chemotherapy and radiotherapy belong to the “drug poison” and “evil poison” from the environment. The clinical feature of oral mucositis, including redness, swelling, ulceration, and pain, are the characteristics of TCM heat toxin syndrome ((Lin et al., 2021)). Heat clearing and detoxicating are classic TCM treatments for heat toxin syndrome (Jin et al., 2009). Therefore, CPMs with the above-mentioned effects have become our ideal option in the prevention and treatment of oral mucositis. The possible mechanisms of CPMs related to their main active components, such as matrine and cockroach extract, mainly attribute to the antioxidant, radical scavenging, anti-inflammatory, antimicrobial, and immunostimulatory properties. More specifically, matrine, as a primary component of Compound Kushen injection, could exert anti-inflammatory effects via lowering the expression of MyD88, NLRP3, and caspase-1, inhibiting the phosphorylation of I-κB α, and affecting the translocation of NF-κB from the cytoplasm to nucleus (Li et al., 2021). Cockroach extract, as the major component of Kangfuxin solution, could relieve the symptoms of oral mucositis by inhibiting the opening of the calcium-dependent potassium channel, promoting granulation tissue growth and angiogenesis, improving the phagocytic ability of macrophages and lymphocytes (Luo et al., 2016). Xihuang capsules, consisting of four ingredients extracted from natural herbs, including *Bovis Calculus artifactus*, *Moschus, Olibanum*, and *Myrrha*, were proved to reduce the levels of TNF-α and IL-6 detected by enzyme-linked immunosorbent assay (ELISA) in patients’ saliva (Dong et al., 2017). Moreover, Yangyin Shengxue Mixture, Kouyanqing Granules, Mailuoning injection, Kangfuxin solution, and Xipayi mouth rinse were also reported to have the effects of promoting microcirculation, reducing local vascular occlusion, and thus accelerating mucosal repair (Wang W. M. et al., 2010; Zhao et al., 2010; Ding and Gu, 2011; Li, 2011; Li and Tan, 2012; Wang and Ma, 2014; Ding and Xu, 2015; Xia et al., 2015; Bai et al., 2017; Wang et al., 2017; Yan et al., 2017). Therefore the mechanism of CPMs in the prevention and treatment of oral mucositis is not only in accordance with the TCM theory, but also related to the anti-inflammatory mechanism of its main active components, and the two complement and promote each other.

The courses of CPMs for the prevention of radiotherapy and chemotherapy-induced oral mucositis were basically the same as that of the chemotherapy or radiotherapy, and the courses for the treatment were about 1–2 weeks. Regarding safety evaluation, five trials reported the occurrence of adverse effects. The most frequently reported adverse effects were gastrointestinal reactions, which involved Zhenhuang pills, Yangyin Shengxue Mixture, Kouyanqing Granules, Compound Kushen injection, and Shuanghua Baihe tablets (Wang W. M. et al., 2010; Xu et al., 2013; Lian et al., 2017; Yan et al., 2017; Zheng et al., 2018). Specifically, Zhenhuang pills would cause loose stools; Yangyin Shengxue Mixture may lead to mild nausea or diarrhea; Kouyanqing granules could cause mild nausea and vomiting; Compound Kushen injection would sometimes induce mild diarrhea and bitter taste; Shuanghua Baihe tablets may bring about mild or moderate gastrectasia reactions. In the TCM theory, Chinese medicines are divided into four properties, including cold, hot, warm, and cool (Yang et al., 2013). Among the main active components of the above-mentioned CPMs, *Rehmannia glutinosa*, *Asparagus*, *Sophora flavescens*, *Coptis chinensis*, *Bezoar*, and *Scutellaria baicalensis* belong to cold or cool Chinese medicines, and these cold or cool components may cause gastrointestinal reactions such as loose stool, diarrhea, and vomiting due to the patients’ asthenia of spleen and stomach, and heavy dampness (Wang W. M. et al., 2010; Xu et al., 2013; Lian et al., 2017; Yan et al., 2017; Zheng et al., 2018). These adverse reactions are all closely related to the patients’ individual “cold” or “heat” constitution, gastrointestinal environment, and the underlying diseases. However, as the adverse effects of CPMs in the experimental groups were all mild and could disappear after stopping drugs, and fewer people were affected as the drug was discontinued, the statistical difference between the control and experimental groups was not clinically meaningful. Therefore, CPMs could still be considered safe in the prevention and treatment of radiotherapy and chemotherapy-induced oral mucositis.

Nevertheless, there are still some limitations in this study. First, as the study only included Chinese and English literature, and most articles relevant to CPMs are published in Chinese, it may lead to potential language bias and citation bias. Second, since most of the original literatures only showed the min-max age range without detailed age grouping, the patients’ age is a major confounding factor, and as the clinical data for each patient in the original RCT studies are inaccessible, it is impossible for this study to further perform subgroup analysis according to the patients’ age. Therefore, age grouping to reduce confounding bias in future studies was recommended. In addition, due to the application of CPMs depends on the identification of their effective chemical components, and the laboratory evidence was still lacking for most of the CPMs, more high-quality laboratory and animal experiments are still needed. Furthermore, due to the limited number of eligible studies and the publication bias, more high-quality, double-blinded, placebo-controlled RCTs are still needed to confirm the efficacy and safety of CPMs for the prevention and treatment of radiotherapy and chemotherapy-induced oral mucositis in future research.

## 5 Conclusion

This systematic review and meta-analysis indicated CPMs were more frequently used in the prevention than the treatment of radiotherapy and chemotherapy-induced oral mucositis. CPMs such as Compound Kushen injection, Kouyanqing Granules, and Shuanghua Baihe tablets may be effective for the prevention of radiotherapy and chemotherapy-induced oral mucositis through reducing the prevalence, relieving the pain level, and decreasing the occurrence of severe oral mucositis. As for the treatment, CPMs such as Xipayi mouth rinse, Sai wei’an, oral ulcer powder, and Kangfuxin solution may be superior in alleviating the symptoms, reducing the cure days than controls like normal saline, vitamin tablets, and antiseptic or anesthetic mouthwash. More high-quality, double-blinded, placebo-controlled RCTs are still warranted to verify and extend the present results to optimize CPMs for the prevention and treatment of radiotherapy and chemotherapy-induced oral mucositis.

## Data Availability

The original contributions presented in the study are included in the article/Supplementary Material, further inquiries can be directed to the corresponding authors.
